# Aquaporin 4 inhibition alters chemokine receptor expression and T cell trafficking

**DOI:** 10.1038/s41598-019-43884-2

**Published:** 2019-05-15

**Authors:** Michael Nicosia, Satoshi Miyairi, Ashley Beavers, George W. Farr, Paul R. McGuirk, Marc F. Pelletier, Anna Valujskikh

**Affiliations:** 1 Department of Inflammation and Immunity, Lerner Research Institute, Cleveland Clinic, Cleveland, Ohio 44195 USA; 2 Aeromics Inc., Cleveland, Ohio 44195 USA

**Keywords:** T cells, Translational immunology

## Abstract

Aquaporins (AQPs) are water channels that mediate a variety of biological processes. However, their role in the immune system is poorly understood. We recently reported that AQP4 is expressed by naïve and memory T cells and that AQP4 blockade with a small molecule inhibitor prolongs murine heart allograft survival at least partially through diminishing T cell activation, proliferation and trafficking. The goal of this study was to determine how AQP4 function impacts T cells in the absence of antigen stimulation. AQP4 inhibition transiently reduced the number of circulating CD4+ and CD8+ T cells in naïve non-transplanted mice in the absence of systemic T cell depletion. Adoptive transfer studies demonstrated T cell intrinsic effect of AQP4 inhibition. AQP4 blockade altered T cell gene and protein expression of chemokine receptors S1PR1 and CCR7, and their master regulator KLF-2, and reduced chemotaxis toward S1P and CCL21. Consistent with the *in vitro* data, *in vivo* AQP4 inhibition reduced T lymphocyte numbers in the lymph nodes with simultaneous accumulation in the liver. Our findings indicate that blocking AQP4 reversibly alters T lymphocyte trafficking pattern. This information can be explored for the treatment of undesirable immune responses in transplant recipients or in patients with autoimmune diseases.

## Introduction

The aquaporins (AQPs) are a family of ubiquitous membrane proteins, arranged as homotetramers that allow the passive passage of water and other small molecules (hydrogen peroxide, ammonia, glycerol etc.) in and out of the cells^1,2^. There are 13 known mammalian AQPs with overlapping expression within various tissues and organs. Broad expression patterns and an array of potential functions confer upon the AQPs a vital role in many processes from water transport and fluid balance to neuroexcitation and metabolism^3^. Recently, the functions of AQPs in immune cells have been demonstrated. Immature dendritic cells express AQP3 and AQP7 and facilitate micropinocytosis mediated antigen uptake^4^. In addition, AQP3 is expressed on T cells and macrophages and is critical for the actin-cytoskeleton remodeling required for chemokine-dependent cell migration^5,6^. AQP9 is an aquaglycerol channel upregulated upon activation of CD8^+^ T cells and is essential for the development of T cell memory^7^.

AQP4 functions exclusively as a water channel and is expressed in renal collection ducts, renal epithelium, brain astrocytes, fast-twitch fibers of skeletal muscle^8^ and cardiomyocytes^3,9,10^. We have recently demonstrated that the use of a small molecule AQP4 inhibitor improves the viability of mouse hearts subjected to prolonged cold ischemic storage^11^. Furthermore, AQP4 inhibition significantly prolongs the survival of fully MHC-mismatched heart allografts. The extended graft survival is associated with decreased activation of donor-specific T cells and reduced early T cell infiltration into the graft^11^. We found that AQP4 is expressed on CD4^+^ and CD8^+^ T cells, and that *in vitro* AQP4 blockade inhibits T cell proliferation. These results suggest that AQP4 inhibitor directly affects T lymphocytes activation, proliferation and trafficking, but the precise roles of AQPs in each of these processes remain to be investigated.

The goal of the current study was to determine the effect of AQP blockade on resting T cells in the absence of antigen-driven responses. We used a small molecule inhibitor of AQP4, AER-270, with a minimal effect on AQP1 and AQP5 channels. Importantly, AER-270 protected AQP4-expressing but not AQP4-deficient T lymphocytes from lysis in a hypoosmotic shock assay. *In vivo* treatment with AQP4 inhibitor transiently reduced the numbers of circulating T cells in naïve non-transplanted B6 mice but did not lead to systemic lymphocyte depletion. Upon adoptive transfer of congenic T cells from AQP4 inhibitor treated mice into untreated hosts, transferred T cells were found in the peripheral blood in far lower numbers than control untreated cells demonstrating that the effect of AQP inhibition is at least partially T cell intrinsic. Furthermore, AQP inhibition altered gene and protein expression of key chemokine receptors involved in T cell circulation, S1PR1 and CCR7, and reduced chemotaxis toward their respective ligands S1P and CCL21. AQP inhibition downregulated the master transcription factor KLF2 that regulates S1PR1 and CCR7 expression resulting in disruption of normal T cell trafficking. Our results suggest that the targeted AQP blockade alters T cell trafficking *in vivo* at least in part via modifying chemokine receptor expression on T cells.

## Material and Methods

### Animals

Male and female C57BL/6J (H-2^b^) [B6 or CD45.2^+^ B6], male B6. SJL-Ptprc^a^ Pepc^b^/BoyJ [CD45.1^+^ B6], and male BALB/cJ (H-2^d^) [BALB/c] mice aged 6–8 weeks, were purchased from The Jackson Laboratories (Bar Harbour, ME). AQP4 knockout (KO) mice in C57BL/6 background were purchased from RIKEN Bioresource Center (Stock no. RBRC04364). All animals were maintained and bred in the pathogen-free facility at the Cleveland Clinic. All procedures involving animals were approved by the Institutional Animal Care and Use Committee at the Cleveland Clinic and all experiments were performed in accordance with the relevant guidelines and regulation.

### Heart transplantation

Vascularised heterotropic cardiac transplantations were performed as previously described^12,13^. BALB/c heart allografts were preserved in University of Wisconsin (UW) solution (320 mOsm; Preservation Solutions Inc., Elkhorn, WI) for 0.5 hours at 4 °C before transplantation into fully MHC-mismatched B6 mice. Rejection was defined as a loss of palpable heartbeat and confirmed with laparotomy.

### AQP inhibitors

A small molecule inhibitor of AQP4, AER-270/271 (Aeromics LLC, Cleveland, Ohio) was identified as previously described^11^. Mice were injected with AER-271 (10 mg/kg i.p) every 6 hours for 2 or 5 days for a total of either 8 or 20 injections. Control mice were injected with PBS at matching time points and did not have either altered T cell levels in the organs observed or altered transplant rejection kinetics. During *in vitro* incubations, and chemotaxis assays 0.25 μM AER-270 was added to the culture media.

### Cell isolation and culture

Splenic T were enriched using negative selection mouse T cell isolation kit from STEMCELL technologies (Vancouver, Canada) to contain >96% of CD3^+^ cells. Purified cell aliquots of 0.5 × 10^6^ were cultured in RPMI (Gibco Life Technologies, Grand Island, NY) supplemented with 10% FBS (Atlanta Biologicals, Lawrenceville, GA), 2 mM L-glutamine, 5 μM 2-beta-mercaptoethanol, 100 U/ml penicillin G sodium and 100 μg/ml streptomycin sulfate or for cultures measuring S1PR1 – serum free HL-1 supplemented with 2mM L-glutamine, 5 μM 2-beta-mercaptoethanol, 100 U/ml penicillin G sodium, 100 μg/ml streptomycin sulfate with or without AER-270 at 0.25 μM for 1, 3, 6 or 12 hours at 37 °C before spinning down the cells and freezing the pellet in liquid nitrogen and storing at −80 °C prior to RNA extraction or stained for chemokine expression prior to flow cytometry.

### Hypoosmotic shock assay

Freshly isolated splenic T cells from either WT or AQP4^−/−^ B6 mice were incubated on a 96 well plate at 5 × 10^5^ cells/well for 30 minutes at 37 °C in RPMI (Gibco Life Technologies, Grand Island, NY) supplemented with 10% FBS, 2 mM L-glutamine, 5 mM 2-beta-mercaptoethanol, 100 U/ml penicillin G sodium, 100 mg/ml streptomycin sulfate with or without 0.25 μM AER-270 or 0.25 μM 4-(Chloromercuri)benzenesulfonic acid sodium salt (PCMB) (Toronto Research Chemicals, Canada). Media was replaced with 100 μl of deionized water to induce hyperosmotic shock, or PBS as controls. After 5 minutes at 37 °C osmolarity was normalized to 300mOSM with 100 ml containing 10 mM calcein-AM (Life Technologies). Plates were returned to 37 °C for 30 minutes and fluorescence was subsequently read using a microplate reader (Wallac-Victor2 Multilabel Counter, Wallac Inc.,Gaithersburg MD) at 485 nm with emission at 530 nm. Data is expressed as calcein fluorescence per well minus mean fluorescence of wells containing media alone.

### Flow cytometry

#### Antibodies

Fluorochrome-conjugated mAbs – anti-CD4, anti-CD8, anti-CD19, anti-CD44, anti-CD45.1, anti-CD45.2, anti-CCR7 (Clone – 4B12, Cat. # -17-1971-63), unconjugated mAbs, anti-S1PR1/mEDG (MAB7089), donkey anti-rat IgG-Biotin (712-066-153), Rat anti-mouse CD16/CD32 (Mouse BD FcBlock, clone – 2.4G2, Cat. #- 553142), and APC-conjugated streptavidin were purchased from BD Biosciences (San Diego, CA), eBioscience (San Diego, CA), R&D (Minneapolis, MN), Jackson ImmunoResearch (West Grove, PA).

Unless specified elsewhere the following protocol applies for all staining protocols. Aliquots of 10^6^ cells were spun down and pelleted and 100 μl of FcBlock - 1:100 in FACS medium, and cells were incubated for 10 mins at room temperature. Dilutions of fluorochrome-conjugated antibodies at 1:500 or 1:1000 were made in FACS medium and then 100 μl was added to the cells before incubating on ice for 30 mins protected from the light. Samples were then centrifuged at 1500 g for 7 mins, the supernatant was decanted and the samples then resuspended in 300 μl PBS. Samples were filtered prior to acquisition of at least 100,000 events per sample on a BD Biosciences LSRII flow cytometer and data was analysed using FlowJo software (Tree Star, Ashland, OR).

#### Sytox staining

Styox Orange (S34861) was purchased from Thermofisher (San Diego, CA). Sytox was added to PBS at a dilution of 1:15000, and 300 μl was added to samples that had already been pre-stained for other surfaced markers and incubated at room temperature for 20 mins protected from the light and then samples ran as normal.

#### Caspase 3 staining

CaspGLOW Fluorescein (88–7003) was purchased from Invitrogen (San Diego, CA). Cells were made up to 10^6^/ml in full RPMI (described above) and aliquots of 300 K cells were taken. 1 μl of CaspGLOW Fluorescein was added to each sample and cells were incubated at 37 °C and 5% CO2 for 30 mins. Samples were then washed twice in wash buffer before staining for other surface molecules.

#### CCR7 staining

Following FcBlock and prior to staining of other surface antigens 100 μl anti-CCR7 1:20 in FACs medium was added to cells and incubated in a water bath at 37 °C protected from the light for 30 minutes. Cells were then washed in PBS and regular staining for additional surface markers followed.

#### S1PR1 staining

The S1PR1 staining protocol was adapted from a previously established protocol^14^. S1PR1 staining requires low serum FACS media (LS-FACS) – 1x PBS pH 7.0–7.5, supplemented with 0.5% FBS, 1 mM EDTA, and 0.05% NaN_3_. 10^6^ T cells were washed in LS-FACS. Anti-S1PR1 at 0.5 mg/ml PBS were diluted 1:10 in low serum FACS + 2% mouse serum (Sigma-M5905) and added 25 μl/sample. Samples were then incubated in a water bath at 37 °C for 90 mins. Samples were then washed twice in LS-FACS and 25 μl Donkey anti-Rat IgG-Biotin, at 1:200 in LS-FACS and 2% mouse serum-preadsorbed for 1 h on ice, was added per sample and samples were incubated on ice for 40 minutes. Cells were washed twice and then resuspended in 25 μl/sample of LS-FACS and 2% mouse serum and 2% normal rat serum (STEMCELL Technologies – Vancouver, Canada) on ice for 30 minutes. Cells were then washed in LS-FACS before staining with 50 μl of 1:100 Steptavidin bound APC, and other surface markers for 30 minutes on ice protected from the light. This was followed by 2 washes in LS-FACS before resuspending cells in 200 μl of LS-FACS, filtering them and then running the samples on the flow cytometer.

### RNA isolation and quantitative real-time PCR analysis

Total RNA was isolated from individual samples by using RNeasy Plus Mini Kit (Qiagen, Hilden Germany). Reverse transcription was performed by using the High-Capacity cDNA Reverse Transcription Kit and quantitative real-time PCR was done on a 7500 Fast Real-Time PCR System instrument using Taqman Fast Universal PCR Master Mix (2x) and No AmpEraseUNG (all from Applied Biosystems, Foster City, CA). Probes and primers were from Taqman gene expression assay reagents (Applied Biosystems). Data were normalized to Mrpl 32 (Mm00777741-sH) RNA amplification and calculated relative to the expression of the target gene in freshly isolated T cells.

### Chemotaxis assay

Freshly isolated splenic T cells were labelled with carboxyfluorescein diacetate, succinimidyl ester (CFSE) as previously published^15^. Briefly, cells were resuspended at 10^7^/ml in PBS supplemented with 0.1% BSA and incubated with 1.25 μmole/L CFSE for 10 min at 37 °C. The stain was stopped with the addition of several volumes of ice cold PBS and then washed twice more in PBS prior to being made up to 2 × 10^6^/ml in RPMI without phenol-red (Gibco Life Technologies, Grand Island, NY) and supplemented with 10% FBS, 2 mM L-glutamine, 5 μM 2-beta-mercaptoethanol, 100 U/ml penicillin G sodium, 100 μg/ml streptomycin sulfate.

Stained cells were then placed in the upper chamber of a transwell plate (Neuroprobe, Gaithersburg, MD) at 10^5^/well, with AER-270 at 0–1 μM. The lower chamber contained either S1P (0–50 mM) or CCL21 (0–1 μg/ml) in RPMI without phenol-red and supplemented with 10% FBS, 2 mM L-glutamine, 5 μM 2-beta-mercaptoethanol, 100 U/ml penicillin G sodium, 100 μg/ml streptomycin sulfate and with the relevant concentrations of AER-270. Once constructed transwell were incubated at 37 °C for 90 mins. After incubation the plate was centrifuged at 2000 g for 7 mins, the supernatant discarded and the cells resuspended in PBS supplemented with 05% SDS. The fluorescence was then read on a microtiter plate reader (Wallac-Victor2 Multilabel Counter, Wallac Inc., Gaithersburg MD) at 485 nm with emission at 530 nm. Serial dilutions of the cell suspension were measured to establish a standard curve relating fluorescence intensity to cell number, and the intensities of test wells were compared to a standard curve and converted into percentage of cells that migrated across the transwell. The relationship between cell number and fluorescence intensity was linear over the measured range.

### Statistical analysis

Heart allograft survival was compared between recipient groups by using Kaplan-Meier analysis. All other results were analyzed by using a one-tailed Student t-test. The difference between groups was considered significant if the p value was <0.05. Unless noted otherwise, the data are presented as mean ± SD values. Total numbers of animals in each experimental group are indicated in respective figure legends.

## Results

### AQP4 inhibition transiently reduces the numbers of circulating T cells

**Figure 1:**
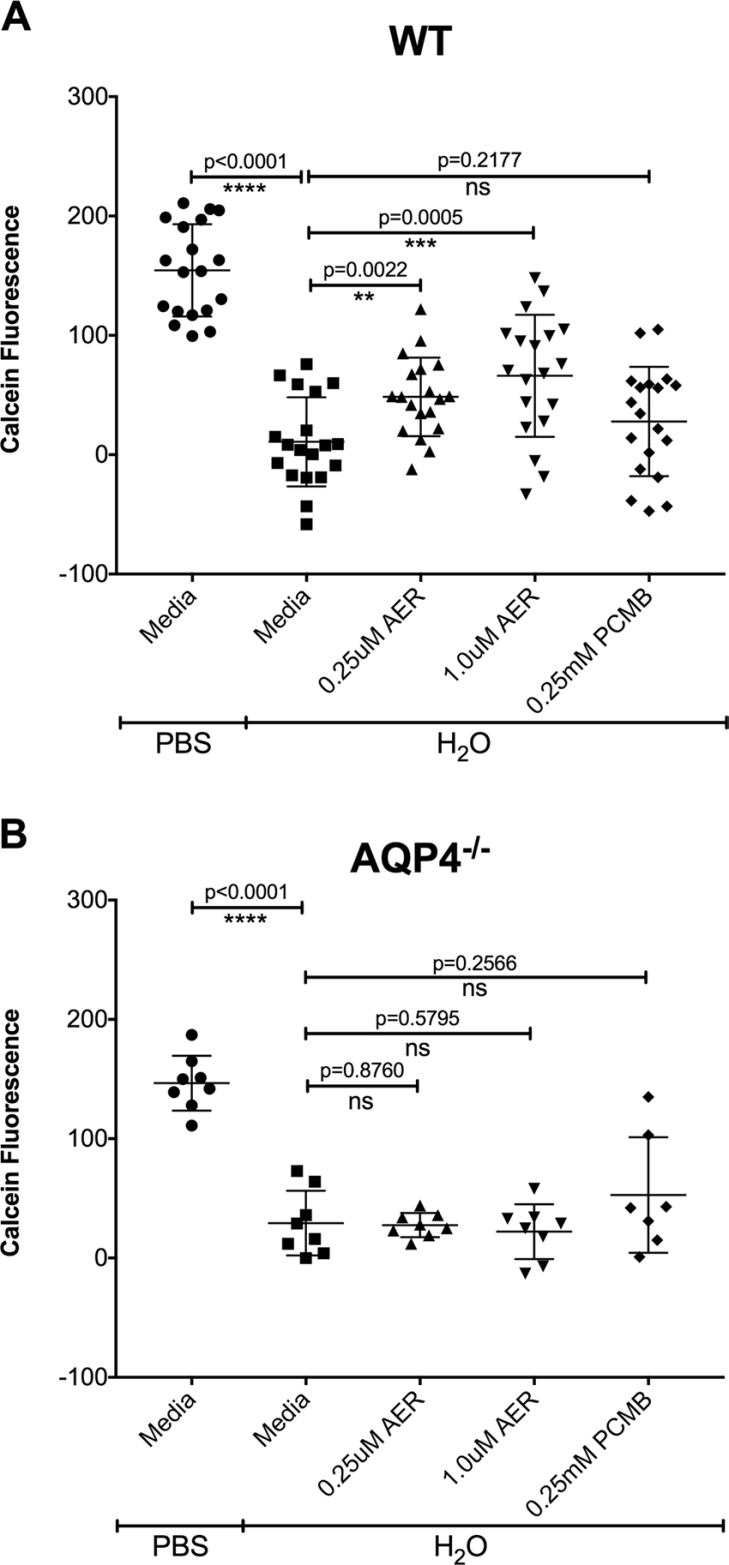
AER-270 protects AQP4-sufficient but not AQP4-deficient T cells from lysis in a water swelling assay. T cells were isolated and purified from either wildtype (**A**) or AQP4^−/−^ (**B**) mice and cultured for 30 minutes in media only or in media containing 0.25 μM AER-270, 1.0 μM AER-270, or 0.25 mM PCMB. After pretreatment, all media was removed, and the cells were exposed to PBS or water for 5 minutes followed by the addition of 100 μl 1x PBS for controls or 2x PBS containing 10 μM calcein-AM. Fluorescence intensity was quantified and the results shown as calcein fluorescence - mean of the fluorescence of wells containing media alone (n = 5–19). *p < 0.05, **p < 0.01, ***p < 0.001, ****p < 0.0001 and ns = no significant change via a one-tailed Student t-test.

To test the functions of AQP4 in the immune cells, we used a small molecule AQP4 inhibitor, AER-270, for *in vitro* studies, or a phosphonate prodrug, AER-271, which is quickly converted into AER-270 *in vivo*^11,16^. We have previously reported that AQP4 is expressed in T lymphocytes and that treatment with AER-270/271 reduces T cell activation and function. This loss of function manifested itself in prolonged graft survival in a robust model of murine heart allograft rejection following peritransplant blockade of AQP4. Futhermore, we demonstrated that peritransplant blockade of AQP4 significantly prolonged graft survival in a robust model of murine heart allograft transplantation. Prolongation of graft survival was characterized by decreased infiltration of T cells into the graft and reduced recall responses to alloantigen^11^. In the current study, we initially tested whether AER-270 inhibits water movement in T lymphocytes. Upon exposure to hypoosmotic shock the rapid influx of water into the cell results in cell swelling and ultimately bursting. Using acetoxymethyl-calcein (calcein-AM) uptake and fluorescence as a quantitative measure of cell viability we found that pre-treatment with AER-270 at 0.25 μM and 1 μM protected T cells from water-induced swelling and lysis (Fig. 1A). Even though T lymphocytes express several members of the AQP family^5,11,17^, pan-AQP inhibitor 4-(Chloromercuri)benzenesulfonic acid sodium salt (PCMB) which targets most AQPs with the exception of AQP4^18^ was not protective in T cells. Furthermore, AER-270 protection was not observed in AQP4-deficient T cells (Fig. 1B), in accordance with recently published data that this reagent does not block other AQPs with high structural homology to AQP4^16^. These data confirm that AER-270 inhibits T cell water transport in AQP4-dependent manner.

**Figure 2:**
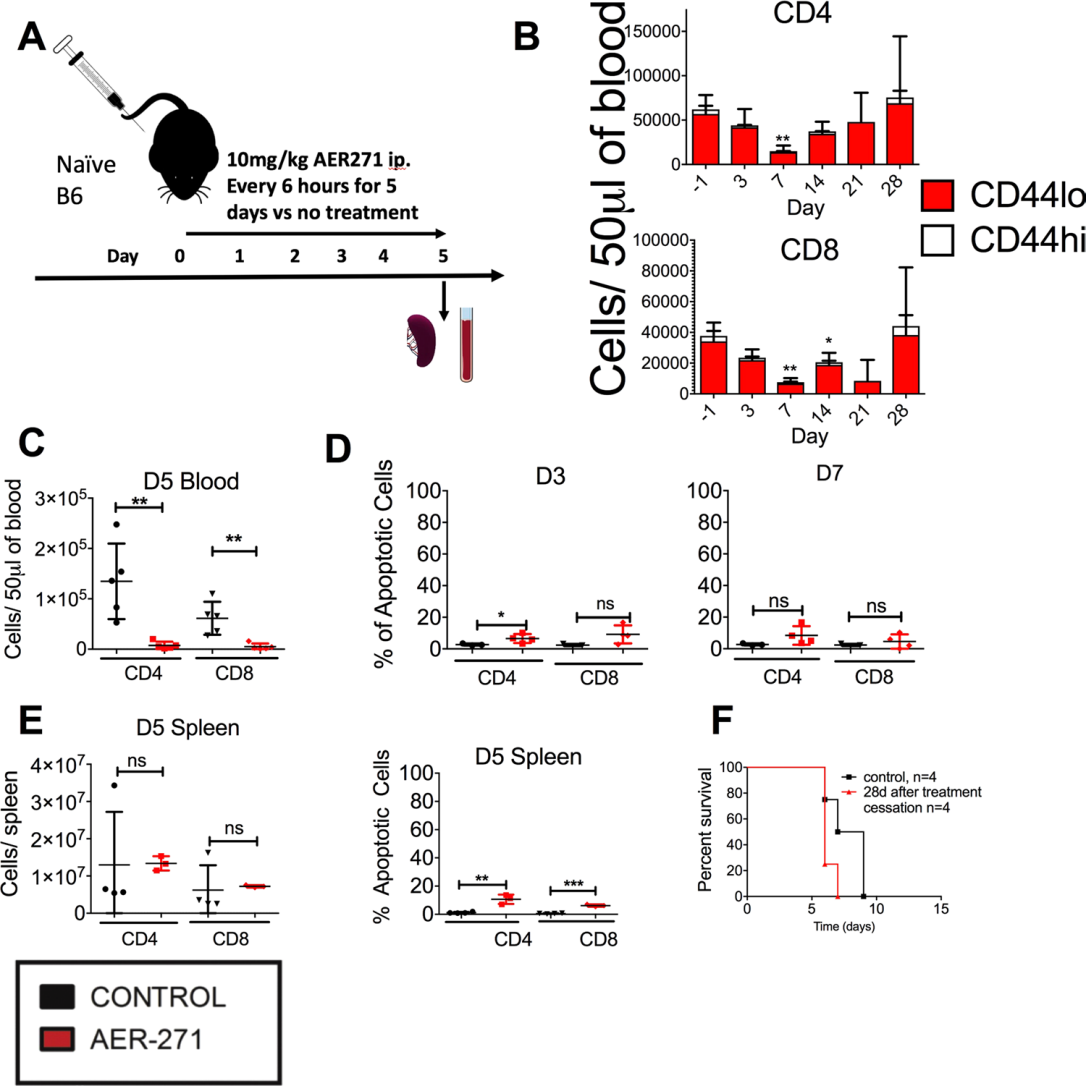
AER-271 treatment reduces numbers of circulating CD4^+^ and CD8^+^ T cells. (**A**) Naïve B6 mice were injected with 10 mg/kg AER-271 or PBS i.p. every 6 hours for 5 days (**A**). The numbers of CD4^+^ (red) and CD8^+^ (blue) T cells in peripheral blood were quantified in PBS treated controls (black) and AER-271 treated (red) (**B**,**C**) (N = 4–6 animals/group. The experiment was performed 4 times with similar results). (**D**) Apoptosis rates of peripheral blood CD4 and CD8 T cells determined by caspase-3 activity staining (N = 3–4 animals/group). (**E**) The numbers and rates of apoptosis of CD4^+^ and CD8^+^ T cells in the spleen (N = 3–4 animals/group). (**F**) B6 mice were treated with AER-271 for 5 days as outlined in (**A**). 28 days after treatment initiation, peripheral blood T cells returned to pretreatment levels, control mice were treated with PBS in line with the AQP4 inhibition protocol and then allowed to rest to the same time point. At that time point, the animals were transplanted with fully MHC-mismatched heterotopic heart allografts from BALB/c donors (n = 4 animals/group). (Heart allograft survival was compared between recipient groups by using Kaplan-Meier analysis. All other results were analyzed by using a one-tailed Student t-test where, *p < 0.05, **p < 0.01, ***p < 0.001, no notation indicates no significant change).

Our previous data highlights AQP inhibition as a potential strategy to target pathogenic alloreactive T cells in transplantation. We sought to determine the effects of AQP inhibition on T cells in the absence of antigen stimulation. Naïve, non-transplanted B6 mice were treated with AER-271 for 5 days (Fig. 2A) and the numbers of CD4^+^ and CD8^+^ T cells were enumerated in peripheral blood. At treatment cessation, AER-271 treated mice had numbers of circulating CD4^+^ and CD8^+^ T cells reduced by 94.5% and 91.8%, respectively, compared to PBS-injected controls (Fig. 2C). The kinetics analysis showed that the reduction in T cell numbers was most prominent on day 7 and had returned to pre-treatment levels by day 21 (Fig. 2B). Limited increase in caspase-3 activity among CD4^+^ or CD8^+^ T cells at either day 3 or day 7 suggested that the reduced T cell numbers are not due to cell death but rather due to an altered distribution (Fig. 2D).

The numbers of CD4^+^ and CD8^+^ T cells in the spleen were not significantly altered in AER-271 treated mice immediately after treatment cessation compared to untreated controls ruling out systemic lymphocyte depletion (Fig. 2E). T cells in the spleen at this time point did exhibit elevated caspase-3 activity after AER-271 treatment compared to controls (Fig. 2E). We next tested the immune competence of T cells returning into circulation after treatment with AER-271. B6 mice were treated with AER-271 for 5 days as outlined in Fig. 2A. 28 days after treatment initiation, peripheral blood T cells returned to pretreatment levels. At that time point, the animals were transplanted with fully MHC-mismatched heterotopic heart allografts from BALB/c donors. The heart allografts were promptly rejected with the kinetics comparable to control B6 mice that have not been treated with AER-271 in the past (MST of 6 d and 8 d, respectively).

### T cell redistribution following AER-271 treatment is partly T cell intrinsic

**Figure 3:**
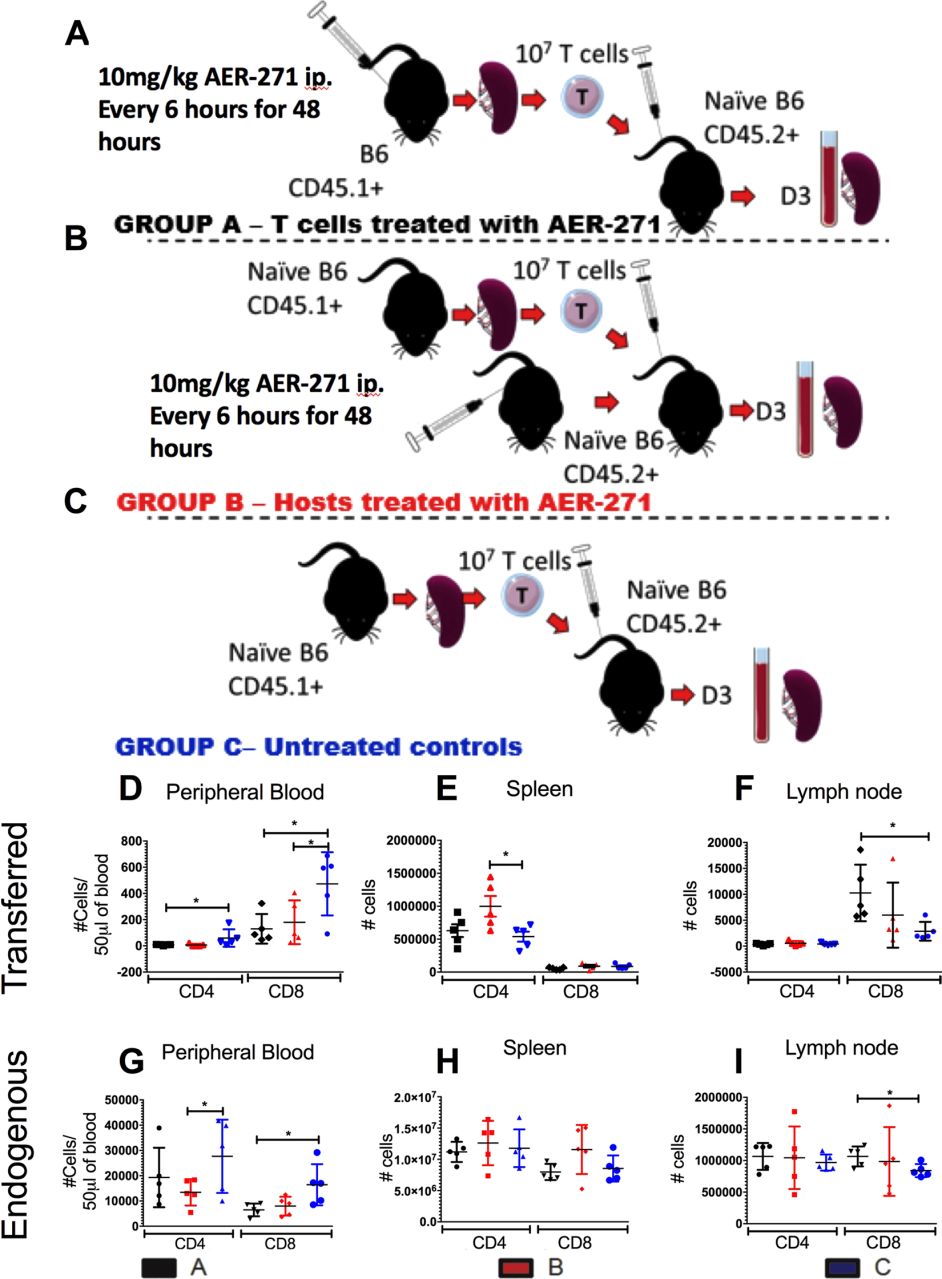
T cell redistribution following AER-271 treatment is partly T cell intrinsic. (**A–C**) Experimental design. 10^7^ isolated T cells from B6.CD45.1^+^ mice were transferred into B6.CD45.2^+^ hosts. Either the T cell donors (group **A**, black) or T cell hosts (group **B**, red) were injected with 10 mg/kg of AER-271 i.p. every 6 h for 48 hours prior to transfer. In control group both T cell donors and hosts were untreated (group **C**, blue). (**D**–**I**) Numbers of transferred (CD45.1^+^) and endogenous (CD45.2^+^) CD4^+^ and CD8^+^ T cells in the blood (**D**,**G**), spleen (**E**,**H**) and inguinal lymph nodes (**F**,**I**) 3 days after T cell transfer (n = 5 animals/group, experiment was repeated 2 times with similar results observed). (*p < 0.05, **p < 0.01, ***p < 0.001, no notation indicates no significant change via a one-tailed Student t-test).

As AQPs are expressed systemically, the effect of AER-271 on non-T cells such as endothelium may account for the observed redistribution of T cells (Fig. 2). To test the effect of AER-271 treatment specifically on T cells, we isolated T cells from congenic B6.CD45.1^+^ mice immediately following 48 hours of treatment with AER-271 and transferred 10^7^ cells into naïve non-transplanted B6.CD45.2^+^ hosts (Fig. 3A). To test the contribution of AQP expressing non-T cells, we isolated and transferred 10^7^ T cells from untreated B6.CD45.1^+^ into naïve non-transplanted B6.CD45.2^+^ hosts that had immediately completed 48 h hour treatment with AER-271 (Fig. 3B). The numbers of transferred CD4^+^ and CD8^+^ T cells were quantified in the blood, spleen and lymph nodes and compared to controls where both B6.CD45.1^+^ T cell donor and B6.CD45.2^+^ host mice were untreated (Fig. 3C).

The previous exposure of T cells to AER-271 treatment altered their pattern of distribution with the numbers of transferred CD4^+^ and CD8^+^ T cells significantly reduced in the blood (Fig. 3D, black). The same tendency, whilst not statistically significant, was observed in group B in which T cells were untreated but the host were pre-treated with AER-271 (Fig. 3D, blue). The endogenous T cells in the peripheral blood were significantly reduced in CD4^+^, and nearing significance in CD8^+^, of group B as anticipated as these cells were directly exposed to AER-271 (Fig. 3G, red). These data suggest that the reduction in numbers of circulating T cells is a partially intrinsic effect. In addition, the numbers of transferred CD4^+^ cells were significantly increased in the spleen of treated hosts in group B suggesting that AER-271 affects spleen cells in a manner that facilitates recruitment/retention of CD4^+^ T cells. There was no statistical significance in the minor variations of endogenous T cells seen in the spleen (Fig. 3H). There was a significant increase in the CD8^+^ T cells found in the lymph nodes of host mice that received T cells from AER-271 treated donors (group A). These data demonstrate that the observed changes in T cell distribution after AER-271 injection are caused by AQP blockade in both T lymphocytes as well as in non-lymphoid cells.

### *In vitro* treatment with AER-270 alters T cell migration caused by S1P and CCR7

Migration of T cells between secondary lymphoid organs (SLOs) and peripheral tissues is crucial for the optimal function of the adaptive immune system. T cell egress and retention in the secondary lymph nodes is dependent on the interactions between cell surface chemokine receptors sphingosine-1-phosphate receptor 1 (S1PR1) and CCR7 and their respective ligands, sphingosine-1-phosphate (S1P) and CCL19/CCL21^19^. Based on observed changes in T cell trafficking we hypothesized that AER-270/271 treatment of T cells leads to altered expression of these key receptors. To test, we isolated T cells from naïve non-transplanted B6 mice and incubated them with or without 0.25 μM AER-270 for up to 12 h. Importantly, the T cell viability was not markedly affected by AER-270 treatment after 1 h or 3 h culture (91.3% vs 94.0% and 87.0% vs 88.6%, compared to untreated controls respectively), was only modestly decreased by 6 h (74.0% vs 82.3%), and reduced by 25% after 12 h (77.25% vs 58.5% live cells for control and AER-270 treatment, respectively).

**Figure 4:**
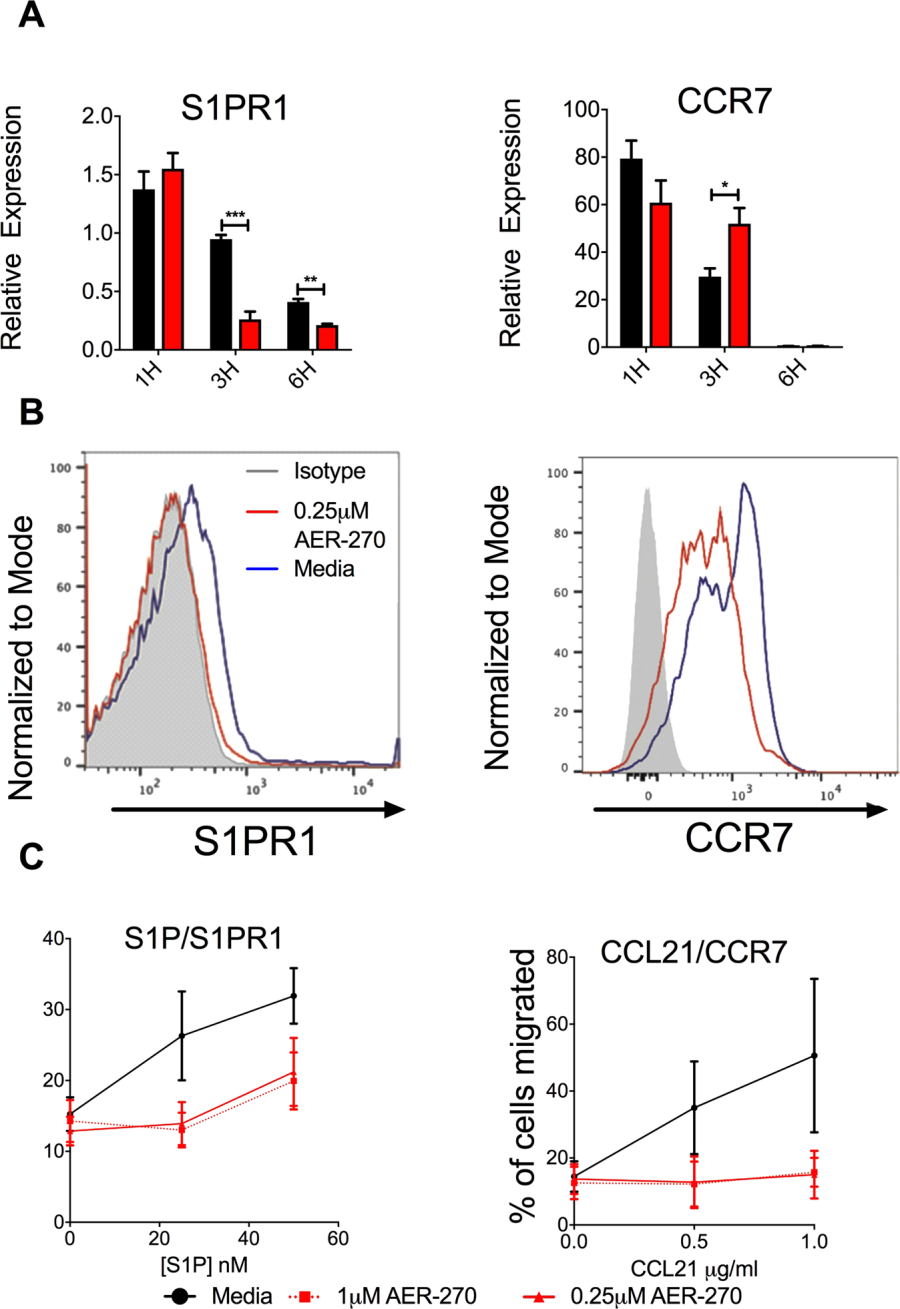
AER-270 treatment alters T cell migration toward S1P and CCL21. T cells were isolated from naive B6 mice and cultured in media only (black) or in media containing 0.25 μM AER-270 (red). (**A**) mRNA expression of S1PR1 and CCR7 by qRT-PCR. Gene expression was normalized against mRNA obtained from freshly isolated, untreated spleen T cells (n = 3–4, and repeated 2–3 times with similar results observed). (**B**) T cells were harvested after 12 h of culture, stained with antibodies against either S1PR1 or CCR7 and analyzed by flow cytometry. Representative histograms are shown (isotype control- grey, media only - blue, AER-270 - red), the experiment was repeated 2–3 times with similar results. (**C**) Freshly isolated T cells were stained with CFSE and 10^5^ cells were then placed in the upper chamber of a transwell plate in media alone (black) or with AER-270 at 0.25 μM (solid red) or AER-270 at 1.0 μM (dashed red). The lower chamber contained either S1P (0–50 nM) or CCL21 (0–1 μg/ml) and the relevant concentrations of AER-270 and placed in an incubator for 90 mins at 37 °C. Cells in the lower chamber were quantified by fluorescence intensity and the results shown as percentage of cells migrated (n = 3–4, and repeated 2–3 times with similar results observed). (*p < 0.05, **p < 0.01, ***p < 0.001, no notation indicates no significant change via a one-tailed Student t-test).

S1PR1 and CCR7 both demonstrated altered gene expression following T cell treatment with AER-270. S1PR1 expression was decreased as early as 3 h after the beginning of treatment with AER-270 compared to untreated controls (Fig. 4A). CCR7 expression was higher in AER-270 treated groups at the 3 h time point and returned to the levels comparable with the controls by 6 h (Fig. 4A). Regardless of changes in mRNA levels, cell surface protein expression for both S1PR1 and CCR7 was decreased following a 12 h AER-270 treatment (Fig. 4B). To assess whether chemokine receptor changes had functional consequences, we tested T cell ability to undergo chemotaxis toward S1P and CCL21 in a transwell system. Treatment with AER-270 diminished T cell chemotaxis in both assays (Fig. 4C) thus supporting the hypothesis that AQP blockade inhibits chemokine-driven T cell trafficking leading to altered T cell distribution.

### AER-270 alters gene expression of master transcription factor KLF2

**Figure 5:**
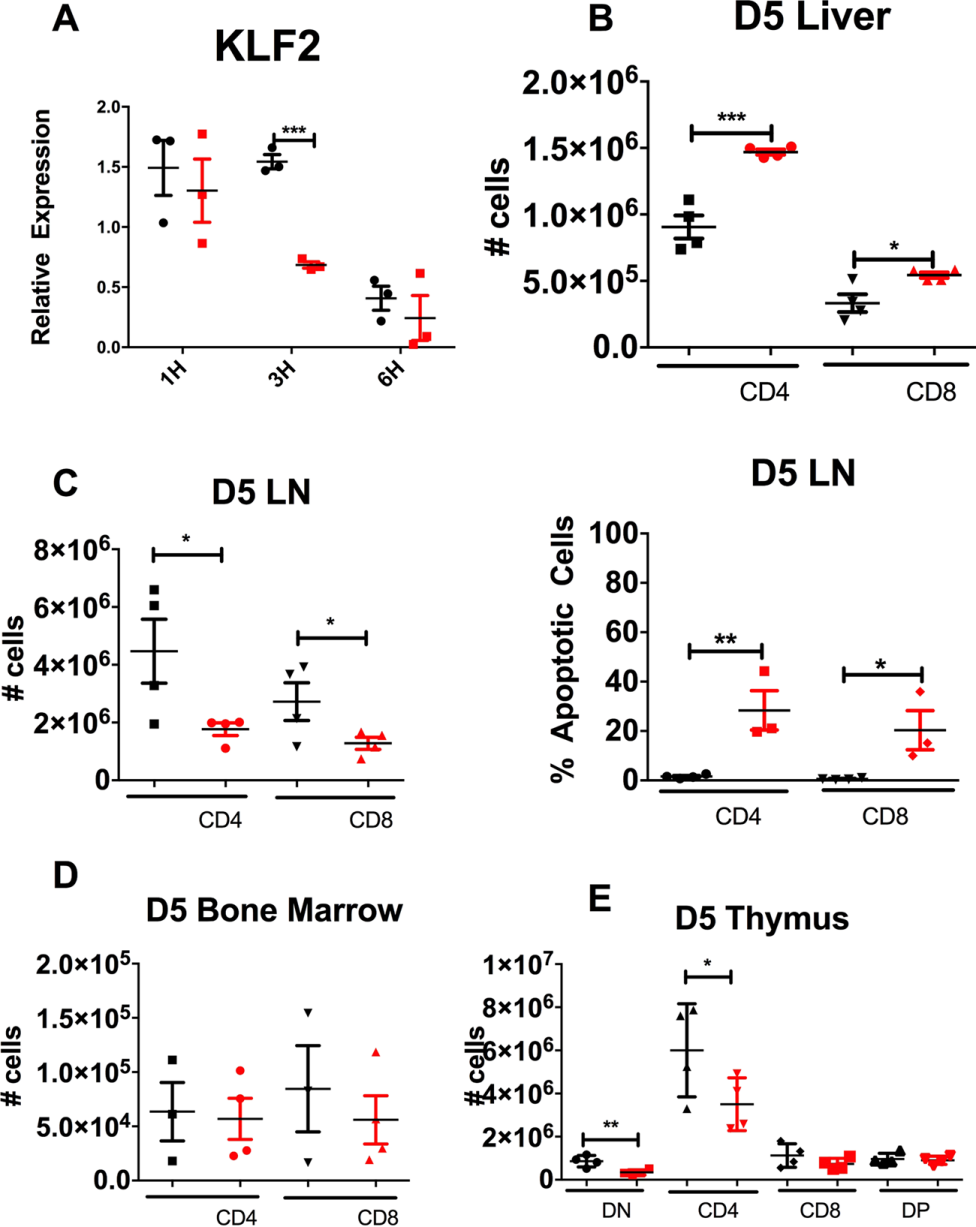
Following AER-271 treatment, the changes in T cell trafficking are consistent with the decreased KLF2 expression. (**A**) mRNA expression of KLF2 by qRT-PCR. T cells were isolated from naive B6 mice and cultured for up to 6 hours in media only (black) or in media containing 0.25 μM AER-270 (red). Gene expression was normalized against mRNA obtained from freshly isolated, untreated spleen T cells (N = 3, repeat experiment performed 3 times with similar results observed). (**B–E**) Naïve B6 mice were injected with 10 mg/kg AER-271 i.p. every 6 hours for 5 days and then sacrificed on day 5. T cell numbers in the liver (**B**), inguinal lymph nodes (**C**), bone marrow (**D**) and the thymus (**E**) were determined by flow cytometry. The analysis of thymocytes included separate enumeration of double negative (DN), CD4^+^ single positive (CD4), CD8^+^ single positive (CD8) and double positive (DP) cells (**E**). AER-271 treated mice (red) were compared to PBS treated controls (black) (N = 4 animal/group). (*p < 0.05, **p < 0.01, ***p < 0.001, no notation indicates no significant change via a one-tailed Student t-test).

As S1PR1 and CCR7 are both regulated by the transcription factor KLF2, we assessed the effect of AER-270 treatment on KLF2 gene expression. *In vitro* treatment with AER-270 decreased KLF2 expression compared to untreated controls (Fig. 5A). Previous reports have shown that decreased KLF2 expression in T cells leads to their accumulation in the liver^20^. Furthermore, the KLF2 regulated receptors control T cell passage through lymph nodes, and bone marrow^21^. We found that both CD4^+^ and CD8^+^ T cells were enriched in the liver following 5 days of AER-271 treatment (Fig. 5B). The T cell numbers in the lymph nodes were significantly decreased in AER-271 treated group compared to controls with the increased proportion of cells undergoing apoptosis (Fig. 5C). AER-271 treatment had no significant effect on the numbers of CD4^+^ or CD8^+^ T cells in the bone marrow (Fig. 5D). In the thymus, AER-271 treated mice had lower numbers of double negative (CD4^−^CD8^−^) precursors and single positive CD4^+^ T cells compared to PBS-treated controls (Fig. 5E). These results are contrary to our expectation that decrease in KLF2 and S1PR1 would prevent mature thymocyte egress and instead suggest that AQP blockade affects thymic cell maturation.

Taken together these data suggest that AER-270/271 treatment reduces the expression of master transcription regulator KLF2, that in turn inhibits CCR7- and S1PR1-dependent chemotaxis which ultimately leads to an altered pattern of distribution characterized by fewer circulating T cells and T cell accumulation in the liver.

## Discussion

Despite the emerging role of AQPs in the immune system, relatively little is known about how transport of water and other small molecule solutes affects immune cell functions and homeostasis. Recently we reported that AQP4 blockade prolongs murine heart allograft survival and decreases T cells infiltration into the graft. However, the direct effect of AQP inhibition on T cell trafficking and anatomical distribution had not been previously addressed. In the current study, we tested the ability of AER-271 to modulate T cell migration in the absence of antigen stimulation.

Multiple studies of structurally different AQPs implicate their importance in cell migration by using gene transfection, knock-out (KO), or siRNA inhibition approaches^22^. AQP-mediated rapid water influx is thought to result in re-stabilization of the membrane in polarized migrating cells^22^. Hydrogen peroxide transport by AQP3 on a variety of cells including T cells and macrophages, provides H_2_O_2_ as a ligand for scaffold protein Cdc42 that is essential for the actin cytoskeletal rearrangements required for chemokine-dependent chemotaxis^5,6^. However, our study is the first demonstration that AQP inhibition of T cells results in diminished expression of chemokine receptors.

The reversible decrease in circulating T cell numbers following AER-271 treatment prompted us to evaluate expression and functions of chemokine receptors regulating T cell migration between secondary lymphoid organs (SLOs) and peripheral blood. S1PR1 expression regulates chemotaxis across lymphatic sinuses towards the natural ligand sphingosine-1-phosphate^23,24^, which is at higher concentrations in the blood and lymph than within the SLOs^25^. The inverse relationship is true of CCR7, with higher levels of CCR7 leading to SLO retention of T cells, as its ligands CCL19/21 are found in the high endothelial venules of the SLOs^26,27^. CCR7^−/−^ T cells have been shown to egress from lymph nodes more quickly than their wild-type counterparts^28^. Consistent with the reduction of circulating T cells in AER-271 treated mice, we found that AQP4 inhibition reduced expression of both S1PR1 and CCR7 on the surface of T cells and inhibited chemotaxis toward S1P and CCL21 (Fig. 4). The transient decrease in cell surface expression of S1PR1 is similar to the effects of FTY720, a superantagonist of S1PR1 which causes receptor internalization and lymphocyte accumulation in SLOs^29^. In contrast, AQP4 inhibitor downregulates S1PR1 by altering its gene expression and therefore AER-271-induced changes in S1PR1 expression and T cell trafficking are slower and longer-lasting than the previously reported effects of FTY720 treatment (Fig. 2 and^29^).

The expression of both S1PR1 and CCR7 is regulated by a zinc-finger transcription factor, KLF2^21,30^. KLF2 is found in a broad range of tissues and responsible for the transcription of vital genes, so that systemic KLF2 deficiency is incompatible with life^31^. KLF2 regulated genes are important for T cell survival, trafficking and function. In particular, T cell KLF2 deficiency results in abnormal patterns of *in vivo* distribution. Our data are consistent with the exclusion of KLF2-deficient T cells from circulation and lymph nodes and accumulation in non-lymphoid compartments such as liver (Fig. 5 and^20^). It remains to be elucidated whether AQP inhibition by AER-270/271 affects other KLF2 expressing cells, or other genes that are regulated by KLF2. For example, the cell surface expression of L-selectin (CD62L), an adhesion molecule regulated by KLF2 and important for T cell SLO entry, was also decreased by AER-270 treatment (data not shown). Our results do not rule out the potential effects of AER-271 treatment on AQP expressing stromal cells or endothelial cells and the expression of adhesion molecules that may further alter lymphocyte trafficking. Furthermore, the link between water movement inhibition and decreased KLF2 expression has yet to be investigated and future studies will focus on the biochemical mechanisms that produce this change.

We observed some variations in the effects of AER-271 treatment on the trafficking of CD4^+^ and CD8^+^ T lymphocytes (Fig. 3). While CD4^+^ and CD8^+^ T cells share expression of many chemokine receptors, it must not be assumed that they use identical migration mechanisms. For instance, lymph node homing by CD4^+^ but not CD8^+^ memory T cells is independent of CCR7^32,33^. CD4^+^ T cells dominate the T cell pool found in the afferent lymph, suggesting that CD8^+^ T cells migrate through lymphatic vessels less efficiently^34,35^. Recently we demonstrated that AQP4 expression is greater in CD8^+^ T cells rather than CD4^+^ T cells^11^. A greater dependency on AQP4 water transport in CD8^+^ T cells may contribute toward the observed variations in migration.

AER-271 treatment increased the proportion of apoptotic T cells in peripheral blood, spleen and lymph nodes (Figs 2 and 5). We have previously reported that one hour after AER-271 injection, the plasma concentration of converted AER-270 has a relatively minor effect on lymphocyte viability^11^. Therefore, it is unlikely that the increases T cell apoptosis is caused by the direct toxicity of the inhibitor. As S1P mediated signaling is required for optimal T cell survival, the observed increase in T cell death may be the consequence of down-modulated S1PR1 expression^14^.

The activity of AER-270 was previously assessed as 52.0 ± 2.0% inhibition of water permeability through AQP4, with minimal effects on AQP1 and AQP5^11^. While T lymphocytes were shown to express several other AQPs such as AQP3, 7 and 9, our data suggest that AQP4 plays the major role in water permeability in resting T cells. Even if AER-270 inhibits water permeability through other members of AQP family, the effect is not sufficient to confer protection in a cell swelling assay. Nevertheless our results do not completely rule out the possibility that AER-270 directly influences other molecular pathways that alter regulating gene expression. The ongoing studies in our lab are focused on investigating whether AER-270 induced changes in water movement are the main cause of altered cell trafficking.

Taken together, our findings provide the first known demonstration that targeted blockade of AQP4 results in decreased KLF2 gene expression and the subsequent decreased expression of key chemokine receptors that mediate T cell migration. This information can be explored for the treatment of undesirable immune responses in transplant recipients or patients with autoimmune diseases.
